# Taking Medicine

**Published:** 1858-07

**Authors:** 


### TAKING MEDICINE.

IF people will guzzle such villanous stuff as castor oil and cod-liver oil, it may be well to know that BORDEN'S *Condensed Milk*, which is coming into such general use, is a very agreeable vehicle, especially for children. The Condensed Milk is simply pure farm-house milk, deprived of a large part of its water, that is, thickened by boiling, as "sugar-water" is.

If a teaspoonful is the dose, mix two teaspoonfuls of the milk with two of water, then beat in the oil, as we "whip eggs," then add two teaspoonfuls of water, and two teaspoonfuls of lemon syrup: children will take this almost as well as they would take milk.

If grown persons will hold their nose, and hold their breath, they may swallow almost any medicine, without scarcely tasting it. Half a dozen pills may be taken at a single swallow, just as easily as one, if they are put in a spoonful of water, the head thrown back, the mouth open, the spoon introduced far back on the tongue, and suddenly tipped up, leaving the sensation of one or two, only, having been swallowed.

